# Development and validation of a short food questionnaire to screen for low protein intake in community-dwelling older adults: The Protein Screener 55+ (Pro^55+^)

**DOI:** 10.1371/journal.pone.0196406

**Published:** 2018-05-23

**Authors:** Hanneke A. H. Wijnhoven, Liset E. M. Elstgeest, Henrica C. W. de Vet, Mary Nicolaou, Marieke B. Snijder, Marjolein Visser

**Affiliations:** 1 Department of Health Sciences, Faculty of Science, Vrije Universiteit Amsterdam, Amsterdam Public Health research institute, Amsterdam, the Netherlands; 2 Department of Epidemiology & Biostatistics, VU University Medical Center, Amsterdam Public Health research institute, Amsterdam, the Netherlands; 3 Department of Public Health, Academic Medical Center, University of Amsterdam, Amsterdam Public Health research institute, Amsterdam, the Netherlands; 4 Department of Clinical Epidemiology, Biostatistics and Bioinformatics, Academic Medical Center, Amsterdam Public Health research institute, Amsterdam, the Netherlands; University of Zurich, SWITZERLAND

## Abstract

In old age, sufficient protein intake is important to preserve muscle mass and function. Around 50% of older adults (65+ y) consumes ≤1.0 g/kg adjusted body weight (BW)/day (d). There is no rapid method available to screen for low protein intake in old age. Therefore, we aimed to develop and validate a short food questionnaire to screen for low protein intake in community-dwelling older adults. We used data of 1348 older men and women (56–101 y) of the LASA study (the Netherlands) to develop the questionnaire and data of 563 older men and women (55–71 y) of the HELIUS study (the Netherlands) for external validation. In both samples, protein intake was measured by the 238-item semi-quantitative HELIUS food frequency questionnaire (FFQ). Multivariable logistic regression analysis was used to predict protein intake ≤1.0 g/kg adjusted BW/d (based on the HELIUS FFQ). Candidate predictor variables were FFQ questions on frequency and amount of intake of specific foods. In both samples, 30% had a protein intake ≤1.0 g/kg adjusted BW/d. Our final model included adjusted body weight and 10 questions on the consumption (amount on average day or frequency in 4 weeks) of: slices of bread (number); glasses of milk (number); meat with warm meal (portion size); cheese (amount and frequency); dairy products (like yoghurt) (frequency); egg(s) (frequency); pasta/noodles (frequency); fish (frequency); and nuts/peanuts (frequency). The area under the receiver operating characteristic curve (AUC) was 0.889 (95% CI 0.870–0.907). The calibration slope was 1.03 (optimal slope 1.00). At a cut-off of ≤0.8 g/kg adjusted BW/d, the AUC was 0.916 (96% CI 0.897–0.936). Applying the regression equation to the HELIUS sample, the AUC was 0.856 (95% CI 0.824–0.888) and the calibration slope 0.92. Regression coefficients were therefore subsequently shrunken by a linear factor 0.92. To conclude, the short food questionnaire (Pro^55+^) can be used to validly screen for protein intake ≤1.0 g/kg adjusted BW/d in community-dwelling older adults. An online version can be found at www.proteinscreener.nl. External validation in other countries is recommended.

## Introduction

An adequate protein intake is considered key in old age to preserve muscle mass and muscle function and thereby physical function [1, 2]. Epidemiological studies among older adults showed that lower protein intake was associated with an increased risk of 1-year weight loss [3], 3-year lean mass loss [4, 5] and that protein intake ≤1.0 grams per kilogram of body weight per day (g/kg BW/d) was associated with more mobility limitations over 6 years of follow-up [6]. Malnutrition is a common problem among older adults [7], and a low protein intake may contribute to this risk [8].

Although the Recommended Dietary Allowance for protein is ≥.8 g/kg BW/d) [9], international expert panels suggest that older adults should consume 1.0–1.2 g/kg BW/d to maintain and regain muscle, based on evidence from epidemiological and short-term metabolic studies on muscle protein synthesis [2, 10]. Among relatively well-functioning older adults from the US, aged 71–80 y, mean protein intake was estimated at 0.9 g/kg BW/d and 66% had a protein intake ≤1.0 g/kg BW/d [6]. Similar mean protein intake was observed among Dutch community-dwelling older adults (mean age 77 y), [11] and other community-dwelling older adults from the US (aged ≥71 y) [12]. In older adults (mean age 70–78 years) participating in trials testing the benefits of protein supplementation, mean protein intake was estimated at 1.0 g/kg BW/d [13–17]. Although a comprehensive overview is lacking, and the prevalence varies by age and study population, these data suggest that at least 50% of general population older adults (65+ y) has a protein intake ≤1.0 g/kg BW/d.

At present, there is no rapid method to screen for low protein intake in older adults. There are several dietary assessment methods available to measure a person’s nutrient intake. These include (extensive) food frequency questionnaires (FFQs), food diaries, 24-hour recalls over multiple days or food weighting records. A major disadvantage of these methods is that they are time consuming to assess and analyze. For this reason, several short food questionnaires have been developed that can classify a person into high or low intake of specific nutrients. Examples are short food questionnaires for fat [18, 19], cholesterol [19], and fiber [20]. A rapid method to screen for low protein intake in older adults can be used in research to screen older adults that could benefit most from protein supplementation. When proven effective interventions are available, a short screening method is also a usefull tool in clinical practice.

This study aimed to develop and validate a short food questionnaire, the so-called Protein Screener 55+ (Pro^55+^), that can be used to screen for protein intake ≤1.0 g/kg adjusted BW/d among community-dwelling adults aged 55 years and older. To enhance its applicability and feasibility in research and clinical practice, our aim was to include a minimum number of questions with a maximum discriminative capacity.

## Materials and methods

### Development sample: Study design and subjects

For the development of the short food questionnaire, we used data of the Longitudinal Aging Study Amsterdam (LASA). LASA is an ongoing cohort study in a representative sample of Dutch older adults aged 55 years and over living in three geographic regions in the Netherlands and started in 1992. The sampling and data collection procedures have been described in detail elsewhere [21, 22]. For the present study, we used cross-sectional data of a LASA side study conducted in 2014–2015 in which data on dietary intake by means of an FFQ were collected. Ethical approval for the LASA study and the side study was given by the Medical Ethics Committee of the VU University Medical Center Amsterdam, and all participants provided written informed consent.

**Fig 1:**
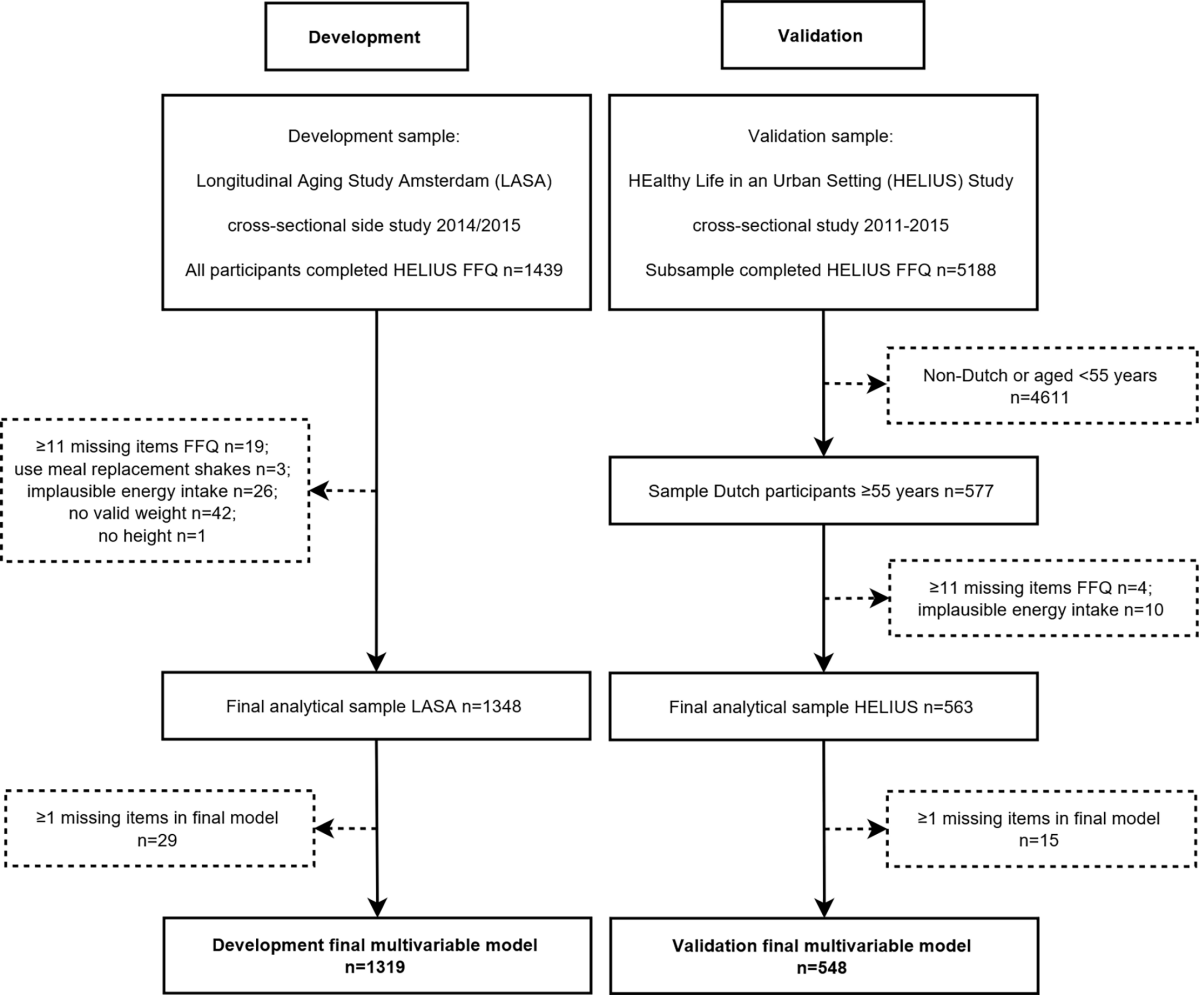
Flow chart describing the selection of the study samples for development and validation of the Protein Screener 55+ (Pro55+).

Of the 1439 participants of the LASA side study, we excluded data of 19 participants with more than 10 missing values on the FFQ questions (due to (accidentally) skipping pages in the paper version, or quitting after item X, or just not completing a (sub) item in the paper version), data of three participants who indicated that they had used meal replacement shakes on more than two weekdays per week, and data of another 26 participants who reported an implausible energy intake (<500 kcal or >3500 kcal for women and <800 kcal or >4000 kcal for men) [23]. Of the 1391 participants left, we excluded data of participants who did not have measured body weight (*n* = 33), had a deviating body weight because of a limb amputation, brace or prosthesis (*n* = 9) or did not have a measured body height (*n* = 1), leaving data from 1348 participants for the analytical sample. In Fig 1, the selection of the development and validation samples is shown.

### Development sample: Measures

Dietary intake was assessed using the 238-item semi-quantitative HELIUS FFQ with a reference period of 4 weeks, that was developed for the HEalthy LIfe in an Urban Setting (HELIUS) study [24]. The HELIUS FFQ was validated using biomarkers: plasma carotenoid levels for fruit and vegetable intake (partial Spearman’s rho = 0.53); and fatty acids measured in the cholesterol-ester fraction of plasma for fish (rho = 0.51) and for high-fat dairy intakes (rho = 0.60) (data available on request). To calculate nutrient intake, all food items were linked to a nutrient database that was based on data of the Dutch Food Composition Table 2011 [25]. Body height and body weight were measured during the medical interview of the regular LASA waves in a standing position wearing light indoor clothing without shoes. Body weight was measured to the nearest 0.1 kg using a calibrated bathroom scale (Seca, model 100, Lameris, Utrecht, the Netherlands). Body height was measured to the nearest 0.001 m using a stadiometer. Corrections have been made to adjust the measured body weight for clothing, shoes or a corset (minus 1 kg for one of those elements and minus 2 kg for more than one), and to adjust the measured body height for shoes (minus 1 cm). Mean body weight was calculated from measured body weight at the waves before (2011–2013) and after (2015–2016) the side study, because body weight was not measured during the side study. When measured body weight from only one of these waves was available, this body weight was used. Measured body height from 2015–2016 was used; when missing, body height from 2011–2013 was used.

### Validation sample: Study design and subjects

For the external validation of the developed short food questionnaire, data of the HELIUS study were used. The HELIUS study is a prospective multi-ethnic cohort study performed in Amsterdam, the Netherlands, and baseline data collection took place in 2011–2015. The sampling and data collection procedures have been described in detail elsewhere [26]. Briefly, data were collected by questionnaire and a physical examination. The population, aged 18–70 years, was randomly sampled—stratified by ethnicity—from the Amsterdam city register. A sub-sample of the HELIUS study was asked to fill in an additional FFQ to measure dietary intake and included 5,188 participants [27]. Ethical approval for the HELIUS study was given by the AMC Ethical Review Board of the Academic Medical Center Amsterdam and all participants provided written informed consent.

For the current validation study, we used cross-sectional baseline data and selected data of Dutch adults aged 55 years and older, resulting in 577 participants. We excluded data of 4 participants with more than 10 missing values on the FFQ questions and data of another 10 participants who reported an implausible energy intake based on the same criteria [23] as in the LASA sample, leaving data from 563 participants for the analytical sample (Fig 1).

### Validation sample: Measures

As in the LASA study, dietary intake was collected using the 238-item semi-quantitative HELIUS FFQ [24, 25]. Body height and body weight were measured in duplicate by trained research assistants during the physical examination with participants wearing light indoor clothing only without shoes. Body weight was measured to the nearest 0.1 kg using a calibrated scale (SECA 877). Body height was measured to the nearest 0.001 m using a portable stadiometer (SECA 217).

### Statistical analyses

Body mass index (BMI) was calculated as body weight (kg) divided by body height squared (m^2^). Educational level was indicated based on the highest education qualification attained, and categorised into 3 groups: low education (never been to school, or elementary schooling only); medium education (secondary schooling or lower/intermediate vocational schooling); and high education (higher vocational schooling or university). Marital status was assessed by a closed question with the following categories: married, living with partner (HELIUS)/registered partnership (LASA), never married, divorced, widowed. Smoking status was categorized into never, past or current smoker.

Protein intake was expressed in grams per kilogram of adjusted body weight per day (g/kg adjusted BW/d). We applied adjusted body weight because in overweight persons, much ‘extra weight’ is adipose tissue, while underweight persons require extra protein to built muscle tissue. Adjusted BMI was calculated for those with a BMI >25 kg/m^2^ (age ≤70 y) or >27 kg/m^2^ (age >70 y) by applying the body weight corresponding to a BMI of respectively 25 or 27 kg/m^2^. For those with a BMI <18.5 kg/m^2^ (age ≤70 y) or <22.0 kg/m^2^ (age >70 y) body weight corresponding to a BMI of respectively 18.5 or 22 kg/m^2^ was applied [12]. Protein intake was classified as either normal/high (>1.0 g/kg adjusted BW/d) or low (≤1.0 g/kg adjusted BW/d) based on increasing evidence and consensus that a protein intake of >1.0 g/kg adjusted BW/d in old age has health benefits [2, 6, 10]. A sensitivity analysis was performed with low protein intake defined as ≤0.8 g/kg adjusted BW/d.

Baseline participant characteristics were presented as mean with standard deviation (± SD), median with interquartile range (IQR) or frequency (%). Statistical analyses were performed using SPSS Statistics version 23 (IBM Corp., Armonk, NY, USA).

### Statistical analyses: Model development in LASA

For the development of the short food questionnaire, we used logistic regression analysis because for use in clinical practice protein intake is dichotomized as low (≤1.0 g/kg adjusted BW/d) or adequate/high (>1.0 g/kg adjusted BW/d). We started to examine the association between all (*n* = 75) questions on *frequency* (in the last 4 weeks) and *amount* (on an average day in the last 4 weeks) of intake of specific foods of the FFQ (candidate predictors) and protein intake ≤1.0 g/kg adjusted BW/d. We chose these 75 candidate predictors because these two basic questions were always posed first in the FFQ, after which more detailed questions (*n* = 163) were asked, on e.g. different subtypes of the foods.

First, the distribution of answers to the 75 questions on frequency and amount of intake of specific foods was examined and recoded if necessary. For example, answers to the questions on frequency of food intake (10 categories) were recoded to 8 categories (<1 d/wk (3 categories merged); 1 d/wk; 2 d/wk; until 7 d/wk) to create a continuous variable with more equally spaced intervals. Answers to other questions were categorized in 2–4 groups when there were too few cases in certain categories to create a continuous variable. In case there was no statistical variation in the answer to a question, the association was not examined. For example, the association with *amount* of food intake was not examined for food products with a low *frequency* of intake. Another example is that of participants who consumed dairy desserts, 81% indicated that they ate one bowl only, resulting in little statistical variation in the amount question on that food product. In case the answer to the question on amount of food intake was missing because this product was not consumed by the respondent in the last 4 weeks, the amount was recoded to the ‘lowest amount category’. The associations between all recoded food variables (frequency and amount) and protein intake ≤1.0 g/kg adjusted BW/d were then examined by univariable logistic regression models, calculating the *P*-values (and Wald statistics) to select the relevant variables for the multivariable model. As the majority of the food variables were associated with protein intake ≤1.0 g/kg adjusted BW/d in the univariable analyses, we applied a strict cut-off of *P*-value <0.01 for inclusion in the multivariable model.

The multivariable analysis started with a full prediction model. A restricted prediction model was then created by using a manual backward stepwise selection approach. First, at each step the variable with the highest *P*-value was omitted until all variables had a *P*-value of <0.01. Second, as we wanted to include a minimum number of questions with a maximum discriminative capacity, the model was further reduced by omitting—step by step—variables with the lowest Wald statistic until the next food item to be removed was an important contributer of protein intake according to a previous study in the general Dutch older population [28]. Third, we removed food items that were less important contributers of protein intake [28]. At each of these three restriction steps, we examined the discriminative capacity of the (restricted) model by the area under the receiver operating characteristic curve (AUC). We added ‘adjusted body weight’ to the final model so that model performance would not depend on body weight.

The performance of the final model was assessed by discrimination, expressed as the AUC, and calibration, visualized in a calibration plot and quantified by the slope of the calibration line. An AUC of 0.5 represents worthless discrimination and 1.0 perfect discrimination. Discrimination of a model is generally considered as good when the AUC is above 0.8. A well calibrated model has a slope of 1.0 [29]. To illustrate the model performance for use in practice, sensitivity, specificity, positive predictive value (PPV), and negative predictive values (NPV) were calculated for three cut-offs of the predicted probability. We additionally examined the performance of the model at a cut-off of ≤0.8 g/kg adjusted BW/d to define low protein intake. This was done by including the food items of the final model in a multivariate regression model with protein intake ≤0.8 g/kg adjusted BW/d as the dependent variable and subsequently calculating the AUC.

### Statistical analyses: Model validation in HELIUS

For the external validation of the final prediction model, we calculated the predicted probability for each subject in the validation sample by applying the regression equation (regression coefficients and intercepts) of the final model from the development sample. The performance of the final model in the validation sample was assessed by discrimination, expressed as the AUC, and calibration, visualized in a calibration plot and quantified by the slope of the calibration line. In case the calibration of the model was considered ‘moderate’ (a slope of <0.95 or >1.05) in the validation sample, we re-calibrated this model by multiplying the estimated regression coefficients from the developed model by a linear shrinkage factor [30]. Sensitivity, specificity, PPV and NPV were calculated based on the re-calibrated model, for three cut-offs of the predicted probability.

## Results

### Sample characteristics

The characteristics of the development and validation samples are shown in Table 1. Participants of HELIUS were somewhat younger (mean age 62 y, SD 4) and higher educated (49% high education) than of LASA (mean age 69 y, SD 9, 30% high education), and mean BMI was slightly lower in HELIUS (26.1 kg/m^2^, SD 4.2) compared to LASA (27.7 kg/m^2^, SD 4.0). An equal percentage was female (52%) and mean intakes of energy, protein, and energy from macronutrients were comparable between the samples. About 30% had a protein intake ≤1.0 g/kg adjusted BW/d in both samples and 11% (LASA) respectively 10% (HELIUS) had an intake ≤0.8 g/kg adjusted BW/d. In both samples, those with a protein intake ≤1.0 g/kg adjusted BW/d were older and had a lower energy intake than those with a protein intake >1.0 g/kg adjusted BW/d. In HELIUS, but not in LASA, women more often had a protein intake ≤1.0 g/kg adjusted BW/d than men.

**Table 1:** 

			Development: LASA			Validation: HELIUS study		
			Total population	Protein intake≤1.0 g/kg adjusted BW/d	Protein intake≥1.0 g/kg adjusted BW/d	Total population	Protein intake≤1.0 g/kg adjusted BW/d	Protein intake≥1.0 g/kg adjusted BW/d
N (%)			1348	409 (30.3)	939 (69.7)	563	167 (29.7)	396 (70.3)
Age, y			69 ± 9	71 ± 9	69 ± 8	62 ± 4	62 ± 4	61 ± 6
% women			52	53	53	52	59	48
Education		% Low	12	15	11	4	7	3
		% Medium	58	54	60	47	26	47
		% High	30	31	29	49	47	50
Marital status		% Married	70	72	66	58	59	54
		% Living with partner	2	2	2	6	7	4
		% Never married	8	8	8	15	14	17
		% Divorced	7	7	7	16	14	18
		% Widowed	13	11	18	5	5	6
BMI, kg/m^2^			27.1 ± 4.3	27.7 ± 4.0	26.9 ± 4.5	26.1 ± 4.2	26.3 ± 4.0	26.0 ± 4.3
Body weight, kg			78.8 ± 14.6	80.9 ± 14.0	77.9 ± 14.8	78.1 ± 14.6	78.6 ± 14.2	77.8 ± 14.8
Smoking	% Never		28	28	28	26	26	27
	% Former		60	60	60	53	53	52
	% Current		12	12	11	21	21	21
*Dietary intake*:								
Energy, kcal			2087 ± 574	1630 ± 384	2286 ± 527	2109 ± 586	1591 ± 373	2327 ± 518
Protein, g/kg adjusted BW/d			1.1 ± 0.3	0.8 ± 0.1	1.3 ± 0.3	1.1 ± 0.3	0.8 ± 0.1	1.3 ± 0.3
Protein, g/d			81 ± 23	58 ± 11	91 ± 20	81 ± 23	57 ± 12	91 ± 19
Protein, % of kcal			15.6 ± 2.6	14.5 ± 2.5	16.1 ± 2.4	15.4 ± 2.6	14.4 ± 2.4	15.8 ± 2.6
Carbohydrate, % of kcal			42.2 ± 6.8	43.7 ± 7.4	41.5 ± 6.4	39.0 ± 6.7	40.4 ± 7.6	38.5 ± 6.2
Fat, % of kcal			32.7 ± 5.9	31.2 ± 6.1	33.3 ± 5.6	34.4 ± 6.1	32.9 ± 6.6	35.0 ± 5.7

Characteristics of the study samples of men and women aged 55+ years by protein intake

Values are means ± SD unless otherwise indicated; BMI, body mass index; BW, body weight

### Steps for developing model

Results of the univariable models are shown in S1 Table. Of the 75 food questions tested, 29 questions (frequency and/or amount) were selected based on their association with protein intake ≤1.0 g/kg adjusted BW/d with a *P*-value <0.01. The AUC for the multivariable prediction model containing all 29 questions was 0.910 (95% CI 0.894–0.927). After omitting variables with the highest *P*-value from this multivariable model, until all remaining variables had a *P*-value of <0.01, the AUC of the restricted model was 0.886 (95% CI 0.867–0.905). This model contained *n* = 16 questions on frequency and/or amount of intake of bread, cheese (on bread), egg, milk, dairy dessert, legumes, pasta, fried potatoes, vegetables, fish, meat, and nuts/peanuts. After further reduction of this model based on the lowest Wald statistic, the AUC of the restricted model was 0.872 (95% CI 0.851–0.893) with *n* = 12 questions. The omitted variables were: ‘amount of intake warm vegetables’; ‘frequency of intake fried potatoes’; ‘frequency of intake legumes’; and ‘frequency of intake meat with warm meal’. We subsequently omitted the question on ‘frequency of vegetable intake’ from the model because vegetable consumption hardly (<4%) contributes to protein intake in the general Dutch older population [28] (AUC restricted model 0.863 (95% CI 0.842–0.885, *n* = 11 questions)). We finally omitted the question on ‘amount of cheese on bread’ from the model because a question on number of slices bread/crackers with cheese was already included in the multivariate model and little discriminative capacity was lost (AUC final restricted model 0.856 (95% CI 0.835–0.878) *n* = 10 questions).

### Final developed model

Our final model included adjusted body weight and 10 questions on the consumption (amount on an average day or frequency in 4 weeks) of: slices of bread (number); glasses of milk (number); meat with warm meal (portion size); cheese (both amount and frequency); dairy products (like yoghurt) (frequency); egg(s) (frequency); pasta/noodles (frequency); fish (frequency); and nuts/peanuts (frequency). The original questions and (recoded) answer categories of this model are shown in S1 Table. All (recoded) predictors and corresponding regression coefficients are shown in Table 2.

**Table 2:** Final model for prediction of protein intake ≤1.0 g/kg adjusted BW/d in community-dwelling men and women aged 55+ years from the development sample (*n* = 1319^1^) and re-calibrated regression coefficients based on application of the model in the validation sample.

(Food) questions	Recoded answer categories^2^	*β*^3^	Se	*W*	*P*-value	Shrunk *β*^4^
Constant		19.361	1.700	129.74	0.000	17.812
**Adjusted body weight, kg**^5^		0.106	0.011	99.565	0.000	0.0974
**Slices of bread on average day in last 4 weeeks**	<3 slices	reference category				
	3 slices	-0.326	0.197	2.75	0.098	-0.300
	4 slices	-1.175	0.219	28.75	0.000	-1.081
	≥5 slices	-2.750	0.358	59.13	0.000	-2.530
**Glasses of milk on average day in last 4 weeks**	<1 glass	reference category				
	1 glass	-0.344	0.179	3.69	0.055	-0.316
	≥2 glasses	-1.681	0.254	43.80	0.000	-1.547
**Portion size meat warm meal on average day in last 4 weeks**	Small portion	reference category				
	Medium portion	-1.326	0.219	36.81	0.000	-1.220
	Large portion	-3.074	0.277	123.08	0.000	-2.828
**Consumption frequency dairy product in 4 last weeks**	Continuous scale: <1 d/wk—7 d/wk	-0.175	0.030	34.56	0.000	-0.161
**Consumption frequency egg(s) in 4 weeks**	<1 d/wk	reference category				
	1 d/wk	-0.256	0.203	1.59	0.208	-0.236
	2 d/wk	-0.636	0.226	7.92	0.005	-0.585
	≥3 d/wk	-1.480	0.262	31.89	0.000	-1.361
**Consumption frequency pasta/noodles in 4 weeks**	≤1 d/4 wk	Reference category				
	2–3 d/4 wk	-0.432	0.228	3.59	0.058	-0.397
	1 d/wk	-0.713	0.220	10.52	0.001	-0.656
	≥2 d/wk	-1.409	0.269	27.54	0.000	-1.296
**Consumption frequencyfish in 4 weeks**	≤1 d/4 wk	reference category				
	2–3 d/4 wk	-0.454	0.230	3.88	0.049	-0.236
	1 d/wk	-0.757	0.215	12.45	0.000	-0.585
	≥2 d/wk	-1.100	0.251	19.24	0.000	-1.361
**Consumption frequencynuts/peanuts in 4 weeks**	Not in 4 wk	reference category				
	1–3 d/4 wk	-0.393	0.216	3.33	0.068	-0.362
	≥1 d/wk	-0.888	0.202	19.31	0.000	-0.817
**Consumption frequency bread, bun, rusk, cracker, etc. with cheese or cheese spread in 4 weeks**	Continuous scale: <1 d/wk—7 d/wk	-0.177	0.033	28.77	0.000	-0.163
**Slices of bread, bun, rusk, cracker, etc. with cheese or cheese spread on average day in last 4 weeks**	≤1 slice	reference category				
	2 slice	-0.654	0.179	13.39	0.000	-0.602
	≥3 slice	-1.214	0.283	18.47	0.000	-1.117

^1^29 participants are not included in final multivariable model because of missing values on one or more questions

^2^The original answer categories were recoded into 8 categories (analyzed as continuous variable) or into 2–4 categories, depending on the distribution of the answers

^3^β, unstandardized regression coefficient, the minus sign means that a higher amount/frequency of food intake is associated with a lower log odds on protein intake < 1.0 g/kg adjusted BW/d

^4^Shrunk *β =* shrunken unstandardized regression coefficients, based on linear shrinkage factor of 0.92 estimated by validation of regression equation in validation sample.

^5^Adjusted BMI was calculated for those with a BMI >25 kg/m2 (age ≤70 y) or >27 kg/m2 (age >70 y) by applying the body weight corresponding to a BMI of respectively 25 or 27 kg/m2. For those with a BMI <18.5 kg/m2 (age ≤70 y) or <22.0 kg/m2 (age >70 y) body weight corresponding to a BMI of respectively 18.5 or 22 kg/m2 was applied [12]. d, day; se, standard error; *W*, Wald statistic; wk, week

### Performance of final model in development sample

**Fig 2:**
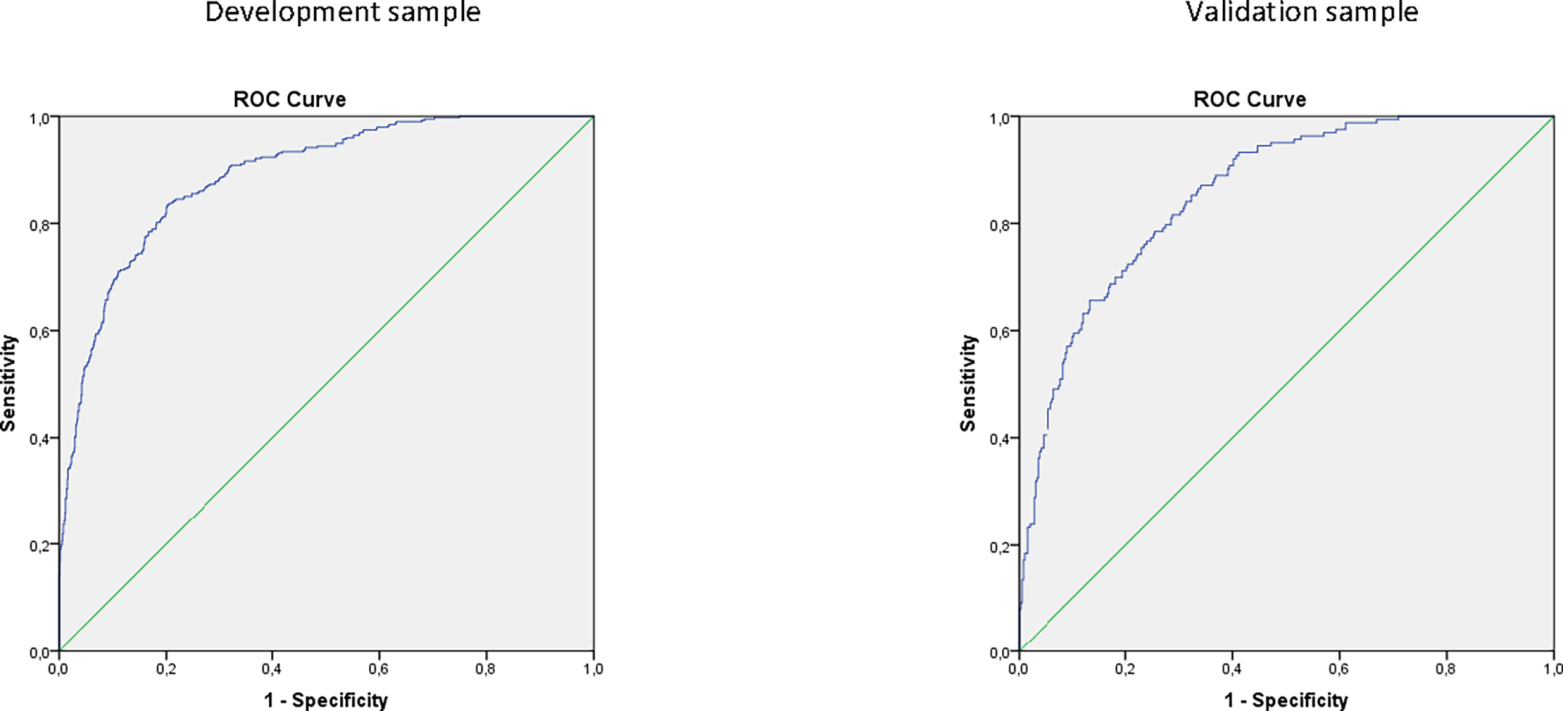
**Receiver-operating characteristic curve of the final model in the development sample (left) and the validation sample (right)**.

**Fig 3:**
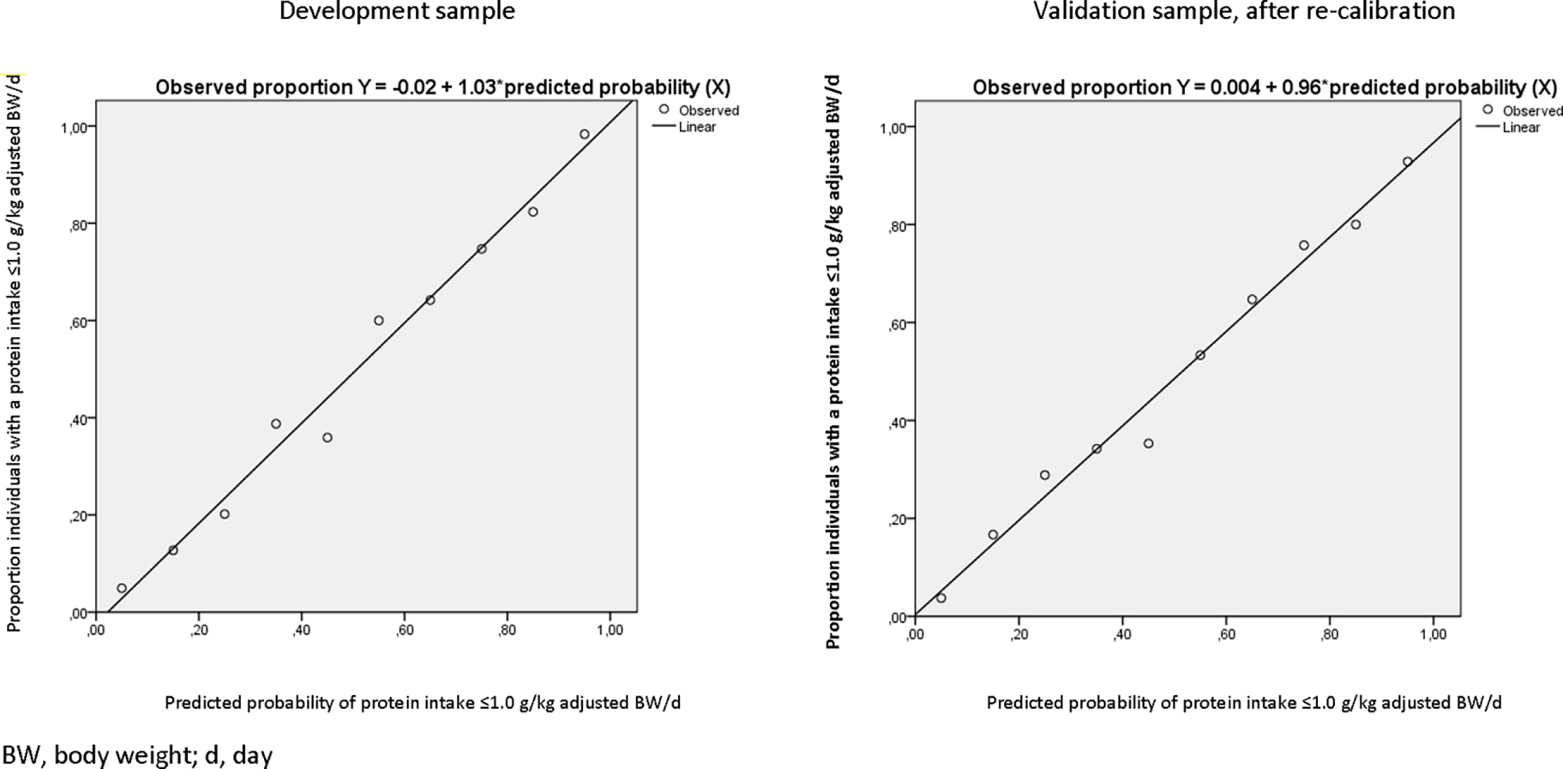
**Calibration plot of the final model in the development sample (left) and the validation sample after re-calibration (right)**.

The AUC of the final model was 0.889 (95% CI 0.870–0.907) (Fig 2). The calibration plot is shown in Fig 3 with a slope of 1.03. In Table 3, the number of cases in each cell, sensitivity, specificity, PPV and NPV are described for different probability cut-offs. For example, 509 out of 1319 (39%) of older persons in the development sample had a predicted probability of >0.3 on having a protein intake ≤1.0 g/kg adjusted BW/d. Of them, 324 (64% = PPV) actually had a protein intake ≤1.0 g/kg adjusted BW/d based on the full FFQ. In total, 394 out of 1319 (30%) older persons in the development sample had a protein intake ≤1.0 g/kg adjusted BW/d based on the full FFQ, of whom 18% (100%-sensitivity) was missed by the prediction model when applying a cut-off of 0.3. When applying a cut-off of ≤0.8 g/kg adjusted BW/d to define low protein intake, the final model performed even better with an AUC of 0.916 (95% CI 0.897–0.936) (data not shown).

**Table 3:** Model performance in the development and validation sample, with protein intake ≤1.0 g/kg adjusted BW/d as the reference standard, at three different cut-off probabilities.

*Development sample (LASA)*							
	Probability:						
	≤0.3	>0.3	≤0.5	>0.5	≤0.7	>0.7	
Protein intake **≥1.0** g/kg adjusted BW/d	*n = 740*	*n = 185*	*n = 839*	*n = 86*	*n = 889*	*n = 36*	
Protein intake **≤1.0** g/kg adjusted BW/d	*n = 70*	*n = 324*	*n = 129*	*n = 265*	*n = 211*	*n = 183*	
**Cut-off probability:**	**>0.30**		**>0.50**		**>0.70**		
Sensitivity, % (95% CI)	82.2 (78.3–85.8)		67.3 (62.5–71.8)		46.4 (41.6–51.4)		
Specificity, % (95% CI)	80.0 (77.3–82.5)		90.7 (88.7–92.5)		96.1 (94.7–97.2)		
Positive predictive value, % (95% CI)	63.7 (59.4–67.8)		75.5 (70.8–79.8)		83.6 (78.3–88.1)		
Negative predictive value, % (95% CI)	91.4 (89.3–93.2)		86.7 (84.4–88.7)		80.8 (78.4–83.1)		
*Vaidation sample (HELIUS)*							
	Probability:						
	≤0.3	>0.3	≤0.5	>0.5	≤0.7		>0.7
Protein intake **≥1.0** g/kg adjusted BW/d	*n = 288*	*n = 98*	*n = 346*	*n = 40*	*n = 372*		*n = 14*
Protein intake **≤1.0** g/kg adjusted BW/d	*n = 36*	*n = 126*	*n = 67*	*n = 95*	*n = 105*		*n = 57*
**Cut-off probability:**	**>0.30**		**>0.50**		**>0.70**		
Sensitivity, % (95% CI)	77.8 (71.0–83.7)		58.6 (51.0–66.0)		35.2 (28.1–42.7)		
Specificity, % (95% CI)	74.6 (70.1–78.8)		89.6 (86.3–92.4)		96.4 (94.2–97.9)		
Positive predictive value, % (95% CI)	56.2 (49.7–62.6)		70.4 (62.3–77.6)		80.3 (70.0–88.4)		
Negative predictive value, % (95% CI)	88.9 (85.2–92.0)		83.8 (80.0–87.1)		78.0 (74.1–81.5)		

BW, body weight; d, day; CI, confidence interval

### Performance of final model in external validation sample

When applying the developed regression equation to the HELIUS data (*n* = 548, 15 persons excluded due to missing data on one of the questions included in the final multivariable model), the slope of the calibration line was 0.92 (Y Calibration = 0.03 + 0.92*linear predictor X). The shrunken regression coefficients, after applying a linear shrinkage factor of 0.92, are shown in Table 2. The calibration plot after re-calibration is depicted in Fig 3 (with a re-calibrated slope of 0.96). The AUC was 0.856 (95% CI 0.824–0.888) when applying the model with the shrunken regression coefficients to the HELIUS data (Fig 2). Model performance as assessed by sensitivity, specificity, PPV and NPV for different probability cut-offs and was slightly poorer compared to the development sample (Table 3). When applying a predicted probability cut-off of >0.3, the PPV was 56% (64% in development sample), and 22% (18% in development sample) of participants with an actual protein intake ≤1.0 g/kg adjusted BW/d was missed by the prediction model.

## Discussion

To our knowledge, this is the first short food questionnaire developed and validated in an external sample to screen for low protein intake in community-dwelling older adults. Our final model included 10 questions on frequency and/or amount of consumption of specific foods, and adjusted body weight. The performance of the model was considered good, based on an AUC of 0.889 and a calibration slope of 1.03 in the development sample and an AUC of 0.856 in the validation sample, indicating that the questionnaire, named the Pro55+ tool, is a valid tool to screen for low protein intake in older adults. A practical online version of the Pro55+ tool can be found at www.proteinscreener.nl.

Despite evidence from prospective epidemiological studies that lower protein intake in old age is associated with greater loss of weight [3], lean mass [4, 5] and a higher incidence of mobility limitations [6], a recent meta-analysis showed there is no evidence from long-term (3 to 24 months) randomized controlled trials that protein supplementation increases lean mass or muscle strength in relatively healthy older adults with a mean protein intake of 1.0 g/kg BW/d [31]. One explanation is that no beneficial effect of extra protein is to be expected when the intake is already sufficient. The short food questionnaire developed in the present study can be used in clinical trials to screen older adults with a protein intake ≤1.0 g/kg adjusted BW/d and examine the beneficial effect of increasing protein intake in this population. If interventions are proven effective, the questionnaire could eventually be used by health care professionals or older adults themselves. The final model also performed well at a cut-off of 0.8 g protein/kg adjusted BW/d, so the questionnaire can also be used to screen for low protein intake at this cut-off. Information on the practical application of the Pro^55+^ can be provided by contacting the authors.

Based on the developed regression equation, a probability score was calculated on a scale from zero to one; with a higher value indicating a higher probability on a protein intake ≤1.0 g/kg adjusted BW/d. Which probability cut-off should be used, depends on the purpose of screening. When it is important to have few false positives value (i.e. protein intake ≤1.0 according to the screener but actual protein intake >1.0), a high probability cut-off like >0.7 should be applied. When it is important not to miss cases with a protein intake ≤1.0 (i.e. protein intake >1.0 according to the screener but actual protein intake ≤1.0), a low probability cut-off like >0.2 should be applied. If the questionnaire is used to describe prevalence rates of protein intake ≤1.0 g/kg adjusted BW/d, a cut-off of >0.3 is recommended as at this point, ‘sensitivity + specificity– 1’ is maximized (data not shown).

This study provided the unique opportunity to develop and validate a short food questionnaire using two different study samples that applied the same 238-tem FFQ. Major other strengths are the nationally representative development sample (LASA), the large sample sizes of both studies, and the availability of measured body weight and body height to calculate adjusted body weight. Another strength is that the validation sample had a different sample selection and slighty different age range compared to the development sample. Since the model performed very well in this external validation sample, the Pro55+ tool seems applicable to all Dutch older adults aged >55 years and older.

Some limitations need to be mentioned. One is that the number of initial candidate predictors was relatively high compared to the number of cases with low protein intake, which could have resulted in an overestimation of the model performance (“overfitting”). However, a major overfitting problem was not observed. The calibration slope of 0.92 in the validation sample indicates that the model slightly underestimates the probability of low protein intake in those with a low risk of a low protein intake and overestimates the probability in those with a high risk of a low protein intake [32]. We therefore applied shrinkage of regression coefficients based on a linear shrinkage factor in the validation sample; this is viewed as an acceptable method to obtain a more valid model for another population [30]. Another limitation is that the reference standard for protein intake ≤1.0 g/kg BW/d was based on the same 238-item FFQ, which may have caused some misclassification. Yet, previous validation studies showed moderate to good agreement between protein intake based on different FFQs compared to several day food records [33, 34], multiple 24-hour recalls [35, 36] or urinary biomarkers [36] although protein intake may be slightly overestimated by the FFQ [37]. As the HELIUS FFQ is even more rigorous than most FFQs, we expect that it is equally or better capable to rank persons with low and high protein intake. Moreover, we would like to emphasize that the short food questionnaire is used for screening purposes only, after which a more extensive assessment of protein intake can take place. In general, as underreporting is a well-known problem in more overweight persons and may as well be a problem in older overweight persons, the questionnaire possibly overestimates lower protein intake, especially in those with a higher BMI. However, this is a known problem for all food questionnaires and not specific for this short questionnaire. The external validity of the Pro^55+^ in other older populations from other countries needs further study, expecially for countries with deviating protein-food consumption habits the questionnaire may need to be adapted and a separate validation study needs to be performed.

To conclude, our developed model to predict protein intake ≤1.0 g/kg adjusted BW/d showed good discrimination and calibration in the development and validation sample. The developed short food questionnaire (the Pro55+) can thus be used to validly screen for protein intake ≤1.0 g/kg adjusted BW/d in community-dwelling older adults. Further external validation in other countries is recommended.

## Supporting information

S1 Table Univariable logistic regression models for prediction of protein intake ≤1.0 g/kg adjusted BW/d in community-dwelling men and women aged 55+ years from the development sample. (DOCX)
