# Supplementary material for: Development and validation of a short food questionnaire to screen for low protein intake in community-dwelling older adults: The Protein Screener 55+ (Pro55+)
Source: PLoS One. 2018 May 23;13(5):e0196406. doi: 10.1371/journal.pone.0196406 (PMC5965846; doi:10.1371/journal.pone.0196406)
Supplement: S1 Table — (DOCX) [file pone.0196406.s001.docx]

**Online Supporting Material**

**Supplemental table 1.** Univariable logistic regression models for prediction of protein intake ≤1.0 g/kg adjusted BW/d in community-dwelling men and women aged 55+ years from the development sample

|  | **Question on:** | **frequency of consumption** | | **consumed amount** |  |
| --- | --- | --- | --- | --- | --- |
|  | **Foods** | **Consumed by:^1^** | **Answer recoded^2^** | **Answer recoded^2^** | **P-value(s)^3^** |
| 1 | Breakfast cereals | 40% | consumed/not consumed | NA^4^ | 0.571 |
| 2 | Ready to use breakfast drink | 4% | NA^4^ | NA^4^ | NA |
| 3 | Dutch risk, crackers | 59% | consumed/not consumed | NA^4^ | 0.488 |
| 4 | Buns, croissants | 69% | 3 categories |  | 0.010/0.001 |
|  |  |  |  | 3 categories | 0.000/0.389 |
| 5 | Slices of bread | 97% | consumed/not consumed |  | 0.000 |
|  |  |  |  | 4 categories | 0.000-0.003 |
| 6 | Gingerbread | 53% | consumed/not consumed | NA^4^ | 0.073 |
| 7 | Butter, halvarine, margarine | 91% | consumed/not consumed | NA^5^ | 0.000 |
| 8 | Cheese on bread/other | 94% | continuous, 8 categories |  | 0.000 |
|  | No. bread/other with cheese |  |  | 3 categories | 0.000/0.000 |
|  | Amount cheese on bread/other |  |  | 3 categories | 0.000/0.001 |
| 9 | Meat on bread/other |  | continuous, 8 categories |  | 0.000 |
|  | No. bread/other with meat |  |  | 4 categories | 0.000-0.267 |
|  | Amount meat on bread/other |  |  | 3 categories | 0.000/0.009 |
| 10 | Peanut butter on bread/other | 44% | consumed/not consumed | NA^4^ | 0.012 |
| 11 | Chocolate on bread/other | 46% | consumed/not consumed | NA^4^ | 0.090 |
| 12 | Sweet on bread/other | 65% | consumed/not consumed | NA^4^ | 0.009 |
| 13 | Spread or salad on bread/other | 41% | consumed/not consumed | NA^4^ | 0.323 |
| 14 | Eggs | 94% | 4 categories |  | 0.000-0.001 |
|  |  |  |  | 2 categories | 0.020 |
| 15 | Milk, buttermilk, soy milk | 66% | 3 categories |  | 0.000/0.034 |
|  |  |  |  | 3 categories | 0.000/0.005 |
| 16 | Yoghurt drink | 15% | NA^4^ | NA^4^ | NA |
| 17 | Chocolate milk | 23% | consumed/not consumed | NA^4^ | 0.869 |
| 18 | Yoghurt, quark, custard, pudding | 81% | Continuous, 8 categories | NA^4^ | 0.000 |
| 19 | Ice cream | 52% | consumed/not consumed | NA^4^ | 0.012 |
| 20 | Whipped cream | 49% | consumed/not consumed | NA^4^ | 0.015 |
| 21 | Soup | 89% | 4 categories | NA^4^ | 0.000-0.330 |
| 22 | Pizza | 33% | consumed/not consumed | NA^4^ | 0.288 |
| 23 | Pancake | 40% | consumed/not consumed | NA^4^ | 0.165 |
| 24 | Pasta | 88% | 4 categories |  | 0.000-0.002 |
|  |  |  |  | 3 categories | 0.000/0.003 |
| 25 | Rice, fried rice | 84% | 3 categories |  | 0.000/0.111 |
|  |  |  |  | 4 categories | 0.000-0.061 |
| 26 | Legumes | 67% | 4 categories |  | 0.000-0.017 |
|  |  |  |  | 4 categories | 0.000-0.038 |
| 27 | Fried potatoes | 63% | 4 categories |  | 0.000-0.029 |
|  |  |  |  | 2 categories | 0.000 |
| 28 | Boiled/smashed/baked potatoes | 94% | continuous, 8 categories |  | 0.000 |
|  |  |  |  | 3 categories | 0.000/0.000 |
| 29 | Warm vegetables | 99% | continuous, 8 categories |  | 0.000 |
|  |  |  |  | 3 categories | 0.000/0.000 |
| 30 | Salad, raw vegetables | 91% | 3 cat. |  | 0.002/0.015 |
|  |  |  |  | 3 categories | 0.000/0.004 |
| 31 | Apple sauce | 55% | consumed/not consumed |  | 0.141 |
| 32 | Shellfish | 33% | 3 categories | NA^4^ | 0.036/0.065 |
| 33 | Fish (no shellfish) | 84% | 4 categories | NA^4^ | 0.000-0.000 |
| 34 | Meat warm meal | 97% | continuous, 8 categories |  | 0.000 |
|  |  |  |  | 3 categories | 0.000/0.000 |
| 42 | Meat replacers | 17% | NA^4^ | NA^4^ | NA |
| 43 | Gravy | 80% | continuous, 8 categories |  | 0.016 |
| 44 | Nuts or seeds with warm meal | 32% | consumed/not consumed | NA^4^ | 0.113 |
| 45 | Cheese with warm meal | 62% | consumed/not consumed |  | 0.009 |
|  |  |  |  | 3 categories | 0.001/0.123 |
| 46 | Cream with warm meal | 39% | consumed/not consumed |  | 0.000 |
| 47 | Warm sauce with warm meal | 72% | consumed/not consumed | NA^4^ | 0.000 |
| 48 | Cold sauce with warm meal | 62% | consumed/not consumed | NA^4^ | 0.000 |
| 51 | Fresh fruit | 97% | continuous, 8 categories |  | 0.002 |
|  |  |  |  | 3 categories | 0.035/0.325 |
| 52 | Wheat biscuit or muesli/grain bar | 11% | NA^3^ |  |  |
| 53 | Pastry | 83% | 3 categories |  | 0.002/0.669 |
| 54 | Cake, large cookie | 81% | 3 categories | NA^4^ | 0.000/0.125 |
| 55 | Small cookie | 86% | continuous, 8 categories | NA^4^ | 0.002 |
| 56 | Candybar | 25% | consumed/not consumed | NA^4^ | 0.053 |
| 57 | Chocolade | 81% | 3 categories | NA^3^ | 0.001/0.442 |
| 58 | Candy | 63% | 3 categories | NA^4^ | 0.015/0.321 |
| 59 | Fried potatoes (snack) | 20% | NA^4^ |  |  |
| 60 | Warm savoury snack | 66% | consumed/not consumed | NA^4^ | 0.077 |
| 61 | Cold salad snack | 24% | consumed/not consumed | NA^4^ | 0.597 |
| 62 | Nuts or peanuts (snack) | 75% | 3 categories | NA^4^ | 0.000/0.006 |
| 63 | Potato chips (snack) | 55% | consumed/not consumed | NA^4^ | 0.570 |
| 64 | Cheese biscuits (snack) | 28% | consumed/not consumed | NA^4^ | 0.092 |
| 65 | Cheese (snack) | 67% | 3 categories | NA^4^ | 0.049/0.611 |
| 66 | Sausage (snack) | 45% | consumed/not consumed | NA^4^ | 0.077 |
| 67 | Toast with cheese/fish/.. (snack) | 59% | 3 categories | NA^4^ | 0.002/0.011 |
| 68 | Coffee | 96% | NA^4^ | 3 categories | 0.031/0.987 |
| 69 | Tea | 88% | NA^4^ | 3 categories | 0.326/0.584 |
| 70 | Water | 73% | NA^4^ | 3 categories | 0.111/0.475 |
| 71 | Fruit juice | 59% | consumed/not consumed | NA^4^ | 0.033 |
| 72 | Fruit drink | 30% | consumed/not consumed | NA^4^ | 0.005 |
| 73 | Lemonade | 20% | NA^4^ | NA^4^ | NA |
| 74 | Soft drink or energy/sports drink | 38% | consumed/not consumed | NA^4^ | 0.047 |
| 75 | Alcoholic beverage | 72% | 3 categories | NA^4^ | 0.038/0.270 |

^1^Percentage of persons that consumed this product in the last 4 weeks; ^2^The original answer categories were recoded into 8 categories (analyzed as continuous variable) or into 2-4 categories, depending on the distribution of the answers; ^3^ *P* value(s) corresponding to univariable models with continuous or categorical variables; all *P* values <0.01 are shaded in grey. ^4^Not analyzed because little variation in distribution; ^5^Not analyzed because of overlap with other questions on amount bread/other.

NA, Not Analyzed; No., number

**Supplemental table 2.** Original questions and (recoded) answer categories for the Protein Screener 55+ (Pro^55+^)

**The following questions are about your dietary habits. It is very important that you give an honest response. We would like to know what you ate or drank in the last 4 weeks (irrespective of week days, weekend days, at home or someplace else). If the last 4 weeks were very special (for example you were sick or you went on a vacation and this had a major influence on your usual diet), please recall the 4 weeks before this period.**

**These questions are about what you eat, not what another person in the household eats.**

| **Original questions 238-item HELIUS FFQ** | **Answer categories questions** | **Recoded answer categories for regression equation** |
| --- | --- | --- |
| ***In the following questions, we ask how much of a food product you ate:*** |  |  |
| 1. In the last 4 weeks, how many slices of bread did you eat on an average day? Mark one answer only. | None / <1; 1; 2; 3; 4; 5; 6; 7; 8; 9; 10; 11; 12; >12 | <3; 3; 4; ≥5 |
| 1. In the last 4 weeks, how many glasses/cups of milk, buttermilk or soy milk did you drink on an average day? Mark one answer only. | None / <1; 1; 2; 3; 4; 5; 6; 7; 8; 9; 10; 11; 12; >12 | <1; 1; ≥2 |
| 1. How much meat did you on average eat on a day that you ate meat with your warm meal in the last 4 weeks? Mark one answer only. | See Picture^1^ | No meat or A (small); B (medium); C-E (large) |
| ***In the following questions, we will ask how often you ate a certain product*** |  |  |
| 1. In the last 4 weeks how often did you yoghurt, quark, milk-based pudding, or soy dessert)? Mark one answer only. | Not in these 4 weeks; 1 d/4 wk; 2-3 d/4 wk; 1 d/wk; 2 d/wk; 3 d/wk; 4 d/wk; 5 d/wk; 6 d/wk; 7 d/wk | Continuous scale with 8 categories (<1 d/wk, 1 day/wk until 7 d/wk) |
| 1. In the last 4 weeks how often did you eat eggs with either your breakfast, lunch, evening meal, as a snack, or in a meal? Mark one answer only. | Not in these 4 weeks; 1 d/4 wk; 2-3 d/4 wk; 1 d/wk; 2 d/wk; 3 d/wk; 4 d/wk; 5 d/wk; 6 d/wk; 7 d/wk | <1 d/wk; 1 d/wk; 2 d/wk; ≥3 d/wk |
| 1. In the last 4 weeks how often did you eat pasta or noodles (like spaghetti, macaroni, lasagna, chow mein, rice-based or wheat-based noodles)? Mark one answer only. | Not in these 4 weeks; 1 d/4 wk; 2-3 d/4 wk; 1 d/wk; 2 d/wk; 3 d/wk; 4 d/wk; 5 d/wk; 6 d/wk; 7 d/wk | ≤1 d/4 wk; 2-3 d/4 wk; 1 d/wk |
| 1. In the last 4 weeks how often did you eat fish with your bread meal, warm meal, or as a snack? (Do NOT include shellfish). Mark one answer only. | Not in these 4 weeks; 1 d/4 wk; 2-3 d/4 wk; 1 d/wk; 2 d/wk; 3 d/wk; 4 d/wk; 5 d/wk; 6 d/wk; 7 d/wk | ≤1 d/ 4 wk; ≥2 d/wk; 2-3 d/4 wk; 1 d/ wk; ≥2 d/wk |
| 1. In the last 4 weeks, how often did you eat nuts or peanuts as a snack? Mark one answer only. | Not in these 4 weeks; 1 d/4 wk; 2-3 d/4 wk; 1 d/wk; 2 d/wk; 3 d/wk; 4 d/wk; 5 d/wk; 6 d/wk; 7 d/wk | Not in 4 wk; 1-3 d/4 wk; ≥1 d/wk |
| 1. In the last 4 weeks how often did you eat cheese or cheese spread on your bread, bun, rusk, cracker, etc.? Mark one answer only. | Not in these 4 weeks; 1 d/4 wk; 2-3 d/4 wk; 1 d/wk; 2 d/wk; 3 d/wk; 4 d/wk; 5 d/wk; 6 d/wk; 7 d/wk | Continuous scale with 8 categories (<1 d/wk, 1 day/wk until 7 d/wk) |
| 1. How many slices of bread, bun, rusk, cracker, etc. with cheese or cheese spread did you on average eat on a day that you ate cheese or cheese spread? Mark one answer only. | None / <1; 1; 2; 3; 4; 5; 6; 7; 8; 9; 10; 11; 12; >12 | ≤1; 2; ≥3 |

d, day; wk, week

**Supplemental Figure 1** ^1^ Picture of portion size of meat, belonging to question 3 of Supplemental table 2.
